# Tumor Digital Masking Allows Precise Patient Triaging: A Study Based on Ki-67 Scoring in Gastrointestinal Stromal Tumors

**DOI:** 10.1155/2018/7807416

**Published:** 2018-09-02

**Authors:** Piotr Lewitowicz, Jaroslaw Matykiewicz, Magdalena Chrapek, Dorota Koziel, Agata Horecka-Lewitowicz, Martyna Gluszek-Osuch, Iwona Wawrzycka, Stanisław Gluszek

**Affiliations:** ^1^Department of Pathology, Faculty of Medicine and Health Sciences, Jan Kochanowski University in Kielce, Kielce, Poland; ^2^Department of Surgery and Surgical Nursing, Faculty of Medicine and Health Sciences, Jan Kochanowski University in Kielce, Kielce, Poland; ^3^Department of General, Oncological and Endocrine Surgery, The Voivodship Hospital in Kielce, Kielce, Poland; ^4^Department of Probability Theory and Statistics, Institute of Mathematics, The Faculty of Mathematics and Natural Sciences, Jan Kochanowski University in Kielce, Kielce, Poland; ^5^Department of Public Health, Faculty of Medicine and Heath Sciences, Jan Kochanowski University in Kielce, Kielce, Poland

## Abstract

**Background:**

Technological advances constantly provide cutting-edge tools that enhance the progress of diagnostic capabilities. Gastrointestinal stromal tumors belong to a family of mesenchymal tumors where patient triaging is still based on traditional criteria such as mitotic count, tumor size, and tumor location. Limitations of the human eye and randomness in choice of area for mitotic figure counting compel us to seek more objective solutions such as digital image analysis. Presently, the labelling of proliferative activity is becoming a routine task amidst many cancers. The purpose of the present study was to compare the traditional method of prediction based on mitotic ratio with digital image analysis of cell cycle-dependent proteins.

**Methods:**

Fifty-seven eligible cases were enrolled. Furthermore, a digital analysis of previously performed whole tissue section immunohistochemical assays was executed. Digital labelling covered both hotspots and not-hotspots equally.

**Results:**

We noted a significant diversity of proliferative activities, and consequently, the results pointed to 6.5% of Ki-67, counted in hotspots, as the optimal cut-off for low–high-grade GIST. ROC analysis (AUC = 0.913; 95% CI: 0.828–0.997, *p* < 0.00001) and odds ratio (OR = 40.0, 95% CI: 6.7–237.3, *p* < 0.0001) pointed to Ki-67 16% as the cut-off for very high-grade (groups 5–6) cases. With help of a tumor digital map, we revealed possible errors resulting from a wrong choice of field for analysis. We confirmed that Ki-67 scores are in line with the level of intracellular metabolism that could be used as the additional biomarker.

**Conclusions:**

Tumor digital masking is very promising solution for repeatable and objective labelling. Software adjustments of nuclear shape, outlines, size, etc. are helpful to omit other Ki-67-positive cells especially small lymphocytes. Our results pointed to Ki-67 as a good biomarker in GIST, but concurrently, we noted significant differences in used digital approaches which could lead to unequivocal results.

## 1. Background

Technological advancement constantly provides cutting-edge tools that enhance the diagnostic capability, although the subcategorisation of many mesenchymal tumors including gastrointestinal stromal tumors (GISTs) is still based on the mitotic count (MC). According to the current Miettinen classification and the European Society of Medical Oncology (ESMO) guidelines, which in fact recapitulates Miettinen's principles, MC is crucial for relevant tumor cross-division to a low- or high-risk recurrence group [1, 2]. An arbitrary cut-off point stated at 5 mitoses per 50 hpf (according to updated 2014 ESMO, it is 5 mm^2^) might be risky in borderline cases and could lead to underestimation. A conventional microscope analysis has natural technical restrictions. There is no doubt it does not allow us to distinguish the field with bigger mitotic activity rendering as the analysed area is random. These limitations of the human eye and the restricted field of view of microscope lens could be countered with more repeatable and efficient tools such as digital image analysis. With time, the labelling of proliferative activity with Ki-67 has become a crucial biomarker. Presently, this is vividly apparent in breast cancer, neuroendocrine tumors (NETs), or brain tumors for which it became a gold standard test hugely influential in tumor grading and gradually replacing MC [3–7]. It could be said that the time of the MC has gone and is gradually replaced by more effective biomarkers. Ki-67 implementation as the predictor encouraged many researchers to study its application in diagnosing other malignancies. A vast spectrum of malignancies has been examined, and notably, most of them confirmed its prognostic value [8–14]. Ki-67 gained a new image after the Cuylen et al. study. They described the unique role of that protein during the cell cycle—namely, the surfactant-like function which allows chromosome separation and its quantitative enhancement during the cell cycle [15]. It gave a foundation for counting not only strong immunohistochemical reactions but also weak ones. On the other hand, the reported differences amidst the immunohistochemical Ki-67 counterpart and counting methodology create new issues with correct tumor categorisation [16–18]. Basically, it means that the achieved scores partially depend on antibody clones or manufacturers, and with the assumption of thresholds, the result could lead to a varied categorisation. It seems, here, we need a digital support; there is no space for random and unrepeatable estimation. The next equally important question was the variable landscape of tumor heterogeneity with the presence of hotspots and many approaches to Ki-67 counting which made a rupture between researchers. Clearly, that could result from the variability of achieved cut-off predictive values and may arise from the absence of standardized, objective, and controlled methods of measurement [18–23]. Focusing on a new approach to heterogeneity especially in the context of hotspot presence reflects the meaning of the most active cell clones in tumors [24, 25]. Employment of objective methods of analysis could help to avoid the subjective pathologist's judgement.

## 2. Objective

The purpose of the present study was tumor digital mapping to estimate the Ki-67 and other cell cycle-dependent proteins as prognostic biomarkers in GIST. Whole slide scanning and digital analysis with detailed mathematical calculations have been planned to reveal intratumoral heterogeneity of proliferative activity.

## 3. Methods

This study has been a natural continuation of our previously published research concerning the significance and the usefulness of GLUT-1, CD9, and CD63 in GIST. The inclusion criteria were previously described in detail [26]. There was no selection bias. This time, we pooled fifty-seven subjects who were eligible for enrolment. In accordance with our previous results, the Miettinen and also the 2012 ESMO guidelines were applied [1, 2]. All cases were reanalysed and recategorised to low- and high-grade GIST. Mitotic count was tabularised as *0–5* and *above 5* mitotic figures per 50 hpf. Simultaneously, immunohistochemical assays with Ki-67, p21, p27, and cyclin D1 were performed and then were scanned and digitally analysed.

### 3.1. Immunohistochemistry

The classical immunohistochemical assays with the use of antibodies against Ki67, p21, p27, and cyclin D1 were performed. All executed assays were fully validated with the intention of in vitro use. The details of used antibodies are presented in Table 1.

All reactions were performed with BenchMark XT (Ventana Medical Systems; Roche Group, Tucson, USA). After the fully automated deparaffinisation and rehydration of the samples, the antigen unmasking processes by CC1 (Ventana Medical Systems; Roche Group, Tucson, USA), incubation with primary antibodies (time and temperature of both antigen retrieval and primary antibody incubation were strictly in accordance with the manufacturer's recommendations), and further routine steps were performed. We used the Ventana ultraView Universal DAB Detection Kit.

### 3.2. Digital Image Analysis of Whole Section Assays

The Hamamatsu NanoZoomer S210 (Hamamatsu®, Hamamatsu City, Shizuoka Pref., Japan) scanner was used for slide scanning. The consequent dedicated digital image analysis was performed using Visiopharm *nuclei plus* application (Visiopharm®, Hoersholm, Denmark). A whole tissue section was scanned which provided great insight into the intratumoral heterogeneity and allowed us to detect the hotspots. We performed double separated calculations for both *hotspot* and *not-hotspot* foci. The whole slide analysis with digitally adjusted, from low to high, magnification allowed pointing to the most active fields (hotspots). The digital templates were stated according to training cycles and settings for intensity of nuclei reaction and simultaneously for nuclei shapes and size. The typical for GIST intertumoral cell shape and size heterogeneity and also spindle vs epithelioid cell type forced a natural correction of previous settings in some cases. Strong nuclear reaction was coded as “strong,” weak nuclear reaction was coded was “weak,” and lack of reaction was coded as “negative.” Digital objects of interest (DOIs) were stated as five 1500 *μ*m × 1500 *μ*m areas corresponding to commonly analysed 50 hpf areas covering ca. 10–11 mm^2^. Importantly, all chosen DOI covered hotspot fields and separately not-hotspots fields for their comparative analysis. All areas in the vicinity of mucosal ulceration and granulation tissue and also rich in stromal Ki-67-positive lymphocytes were rigorously excluded to avoid the falsely raised results. Both weak and strong nuclear reactions were coded as positive. To depict intratumoral heterogeneity of proliferative activity, all calculations were done as follows: the hotspot-positive ratio (HSPR%) depicts the ratio of the all-positive cells counted in hotspots to all analysed tumor cells in DOI; similarly, the not-hotspot-positive ratio (nHSPR%) corresponds to the ratio of the all-positive cells counted in not-hotspots to all analysed cells in DOI.

### 3.3. Statistical Methods

Quantitative data was reported as minimum, maximum, median, lower (Q1), and upper (Q3) quartile (in the case of nonnormal distributions) or as the means and standard deviations. Categorical data was expressed as number and percentage distributions. The chi-square test or Fisher's exact test was applied to compare proportions while the Mann-Whitney test or Kruskal-Wallis test was used to compare distributions of continuous variables. Correlations between continuous variables were assessed by Spearman's rank correlation coefficient. The receiver operating characteristic (ROC) curve analysis was performed to test the ability of analysed variables to distinguish between low (≤3) and high (>3) ESMO. The area under the ROC curve (AUC) with 95% confidence interval (95% CI) was estimated, and the optimal cut-off values were determined. The odds ratios (OR) with 95% CI were also calculated. The recurrence-free survival (RFS) period in two groups was compared by a log-rank test. A two-tailed *p* value < 0.05 was considered as statistically significant. All statistical analyses were performed using R (version 3.1.2; The R Foundation for Statistical Computing, Vienna, Austria) and Statistica (StatSoft Inc., 2014, version 12).

## 4. Results

The average age of patients was 62.2 years (ranging from 31to 89; st. dev. 13.8). The average quantity of analysed cells was 12,567 per singular ROI (1500 *μ*m × 1500 *μ*m area), viz., 5585 cells per 1 mm^2^. All general characteristics of patients and tumors are depicted in Table 2. Moreover, in addition to current calculations, the previously studied CD63 and GLUT-1 were included as well.

Reaching a Ki-67 median value of 6.5% strongly separated the high- and low-grade tumors (*p* = 0.0006) according Miettinen's classification and has been in accordance with the percentage score results. As presented in Table 2, the HSPR% median follows traditional MC (*p* = 0.0009) and also all other calculations of Ki-67 (*p* < 0.0001). Interestingly, the number of analysed cells differs between analysed tumors making labelling restricted only to the area that is potentially wrong (*p* = 0.002). While looking for a promising Ki-67 cut-off, we observed that 16% HSPR% allows for the diversification of very high-grade GIST (5–6 groups) (AUC = 0.913; 95% CI: 0.828–0.997, *p* < 0.00001). Relevantly, Ki-67 in HSPR% > 16% indicates the statistically significant chance for group 4+ (5–6 groups) (OR = 40.0, 95% CI: 6.7–237.3, *p* < 0.0001).

**Figure 1:**
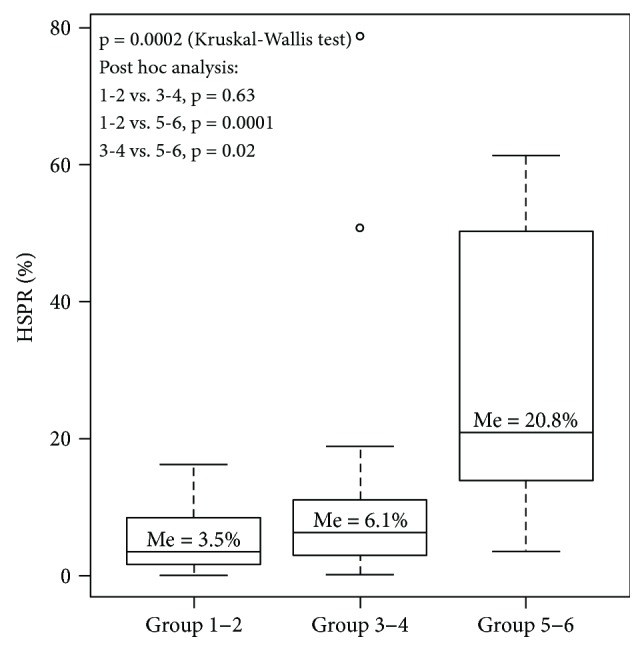
Tercile analysis depicting progress of the Ki-67 score with higher risk of recurrence.

Simultaneously, a tercile analysis splitting patients into three groups was undertaken. Medians at 3.5% and 10% were extracted as follows: low-risk GIST (*n* = 27), moderate- to high-risk tumors (*n* = 17), and very high-risk tumors (*n* = 13)—here we observed a problem with accurate separation of low and moderate GISTs (Figure 1).

**Figure 2:**
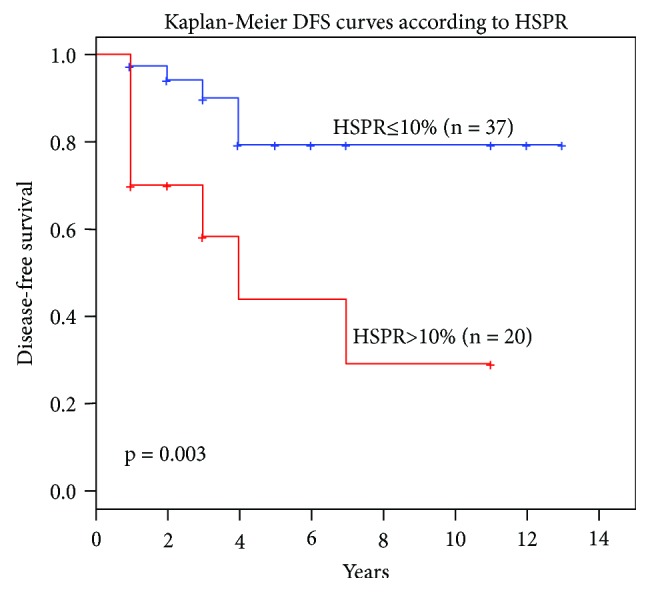
The Kaplan-Meier diagram for RFS and 10% Ki-67 cut-off value.

We compared recurrence-free survival period (RFS) with the 6.5% median by log-rank test (*p* = 0.20), and collaterally, we calculated a tercile analysis for median thresholds at 3.5% and 10%. A 10% cut-off separating 3–4 and 5–6 groups strongly correlated with RFS (*p* = 0.003) which is presented in Figure 2.

**Figure 3:**
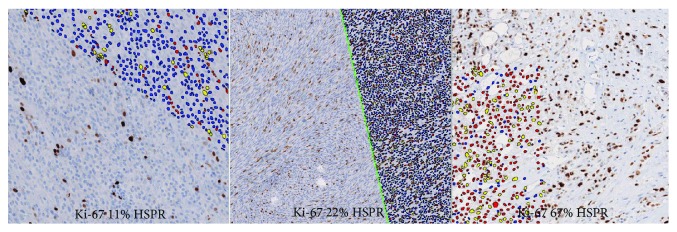
A compilation of Ki-67 labelling with concurrent digital masking. Yellow dots represent strong Ki-67 expression, red dots represent weak expression and blue points correspond to the negative results.

The next task was to face the strong nuclear reaction paradigm. To estimate the impact on weak and strong nuclear reactions the Spearman rank analysis was applied.  Figure 3 illustrates the digital map covering the slide scan. The results showed a strong positive correlation of HSPR% with all items reached by digital analysis. The highest *r* values (0.94 and 0.93) were obtained for total (weak and strong as well) positive cells and selectively only for weak nuclear reactions, respectively.

A comparative analysis of cyclin D1, p21, and p27 with Ki-67 was performed to unveil a biomarker power. Unsurprisingly, the expression level of cyclin D1, p21, and p27 followed the progress of tumor malignancy, although Ki-67 HSPR% reached the most significant meaning (AUC 0.913, *p* < 0001) (Table 3). The same results were achieved by splitting MC into two groups: 0 to 5 mitoses and above 5 mitoses (for Ki-67 *p* < 0.0001).

## 5. Discussion

In the present paper, our attention has been focused on the Ki67 labelling as a standalone biomarker. There are numerous attempts at Ki-67 labelling, and the conclusions always remain consistent. It seems that the diagnostic relevance of Ki-67 could outperform traditional MC and is closer to becoming the routine biomarker in mesenchymal tumors as well. Cuylen et al.'s results helped us to align the meaning of the immunohistochemical nuclear strong and weak reactions which was disputed in the past [15]. Indeed, a very strong nuclear reaction corresponds to the late-phase cell cycle and could depict the vicinity of the mitotic figure. Our results did not confirm the key role of strong reactions; instead, we noted a stronger impact of the total positive reactions. The digital analysis provided us with a very good solution on precise extraction weak/strong reaction, then accurate counting, and finally precise mathematical calculations. It helped us to debunk a myth of strong reaction advantage. An outright indication of a sharp cut-off value for mesenchymal tumors could impose a risk of underestimation or overestimation. Our relatively small cohort allowed us to achieve a sharp cut-off at 6.5% to extract the high-risk tumors according to Miettinen's principles. It is intelligible that the final score could be falsified by stromal inflammatory or other Ki67-positive cells, so perhaps a frame construction with a slender grey zone would prove to be of better use. Obviously, a full cut-off value implementation needs much more numerical cohorts and working group consensus. PubMed-available data concerning GIST and Ki-67 are seriously diversified especially in methodology used. The results published by Liang et al. stated a 5% cut-off as biomarker of a worse outcome. That discrepancy with our data could result from a microarray technique with no hotspot extraction and counting with the naked eye [27]. Belev et al. and Kemmerling et al. also suggested 5% cut-off but the authors analysed only 100 and 1000 cells [28, 29]. Furthermore, Basilio-de-Oliveira and Pannain pointed to a threshold at 134.8 Ki-67-positive cells per mm^2^ as a good predictor of outcome [30]. Zhao et al. applied two thresholds at 5% and 8%, and they reached similar conclusions to ours; however, they solely analysed hotspots. The natural restriction of this study was the use of the microarray technique [31]. Basically, the available published data concerning Ki-67 scores are similar or slightly differ from ours. This requires a short explanation. We have found papers where calculations were based on completely different methodologies such as a cost-saving microarray, covering only hotspots or randomly chosen areas counting only strong reaction or analysing a small cell number. The implementation of so many various methods was bound to lead to various results. Firstly, we designed counting in line with Miettinen rules—on 5 defined areas covering 10–11 mm^2^; secondly, an average above 60,000 cells in all cases was analysed; and finally, we made a diversification between strong reactions, weak reactions, and hotspot and not-hotspot foci. In brief, we applied the whole slide digital assays with hotspot field scoring which was the reason behind the differences. It appears that the microarray could seriously contribute to interstudy discrepancies. The rhetorical question is the best answer—does 1.5–2 mm diameter core tissue truly correspond to real tumor complexity including heterogeneity?

The next question was the choice of field for analysis especially in the light of meaning of hotspots and randomly chosen areas. There is no global consensus on the principles of labelling. In the other words, automated counting in the hotspot fields seems to be the best repeatable solution. In the fresh paper of Thakur et al., what they applied was similar to our methodology, meaning whole slide scanning with hotspots extracted as an unbiased approach [18]. The issue of Ki-67 clones used in immunohistochemistry had been already thought of in the last decade, where MIB-1 clones were presented as one of the highest sensitivities for the Ki-67 antigen. Next, papers covering the newest Ki-67 clones reported comparable concordance between the MIB-1 clone and the 30-9 clone [16, 17].

We observed that both HSPR% and nHSPR strongly correlated with the MC and malignancy levels, but concurrently, the quantitated significant difference (*p* < 0.0001) between HSPR% and nHSPR% was noted (Table 2 depicts huge differences between groups). It has the absolute meaning in the formulation of precise cut-off values. That is why we emphasise using a digital solution, whole slide scanning, and choosing only hot fields to label. That will be relevant for patient triaging based on Ki-67 cut-off values in the future. In light of Miettinen's classification, Ki-67 could become a promising biomarker. Our experience has taught us to keep a distance from prediction based on MC in cases when just a small biopsy had been taken; thus, we previously tried to focus our attention on other biomarkers [26, 31–34]. In a very recent paper by Liu et al. covering 1022 patients, the cut-off values were stated at 6% which is very close our data, although some methodological differences exist between us [35]. The most newly published paper by Sugita et al. applied a methodology similar to ours reaching an 8% score for whole slide scanning and Ki-67 labelling. The authors concluded that whole slide analysis raises the Ki-67 score in high-grade tumors to 8% and assume our study in hotspots could reach 10% or more [36].

However, a clinical oncology light is obligatorily focused on Ki-67 as a crucial biomarker; no others exist that are less efficient. Phosphohistone H3 (pHH3) was widely tested amidst many cancers as a sensitive marker for mitotic figure detection and could be seen as a successor of traditionally scored MC in the close future. Interestingly, its significance was highlighted also in many types of malignancies where the mitotic index is not evaluated routinely. In many papers, the pHH3 ratio was presented as a highly prognostic biomarker [37–42]. In that light, GISTs, as the tumors where categorisation is fundamentally based on MC, have been also researched. If it was assumed that chromatin condensation with positive pHH3 IHC reaction corresponds to the recent stage of mitosis, the final pHH3/MC score must by higher over traditional MC. The authors involved in that issue reported a necessity of reclassifying tumors according Miettinen's rules because of the raised number of pHH3-positive cells than the original MC [43–45]. That is why immunohistochemical and digital support outperforms traditional manual counting of mitoses.

While we did not find the correlation with tumor measurements and tumor location, we noted progress of proliferative activity with intracellular metabolism-dependent proteins, namely, CD63 and GLUT-1. Why the achieved Ki-67 scores between men and women are higher (*p* < 0.05) remains unclear. Assuming absolute lack of selection bias, we suppose that it could result from cohort size.

The comparative analysis showed the absolute Ki-67 prevalence over other cell cycle proteins as a clinically useful biomarker. There is a rising number of digital solutions, but a question regarding the employment of Ki-67 scoring remains unanswered. Our experience does not leave any doubts—digital masking of a large tumor area instead of small-field microscope photography allows us to extract the most active fields. The necessary time for analysis including scanning time and image analysis was close to 10 minutes making this way of scoring both precise and time saving while providing a repeatable and comparable solution.

## 6. Conclusion

Tumor digital masking is a promising solution for repeatable and objective labelling. Software adjustments of nuclear shape, outlines, size, etc. are helpful to omit other Ki-67-positive cells especially small lymphocytes. Moreover, dedicated software allows precise mathematical calculations making results more accurate.

Our results point to Ki-67 as a good biomarker in GIST, but common utilization of Ki-67 should proceed after establishing the guideless for cell counting and finally after larger studies have been conducted in order to reach a consensus of the cut-off value. Lately, published papers present similar results although they were based on different digital applications which proves the advantage of digital labelling.

## Figures and Tables

**Table 1:** The detailed characteristics of used antibodies.

	Clone	Catalogue number	Dilution	Type of antibody	Manufacturer
Ki-67	30-9	790-4286	Ready to use	Rabbit monoclonal mouse	Ventana Medical Systems; Roche Group, Tucson, USA
p21 WAF1	DCS-60.2	790-4286	Ready to use	Mouse monoclonal mouse	Cell Marque, Rocklin, USA
p27Kip1	SX53G8	760-4268	Ready to use	Mouse monoclonal mouse	Ventana Medical Systems; Roche Group, Tucson, USA
Cyclin D1	SP4-R	790-4508	Ready to use	Rabbit monoclonal mouse	Ventana Medical Systems; Roche Group, Tucson, USA

**Table 2:** The tercile analysis for low-/moderate-/high-grade GIST according to Ki-67 HSPR%.

Characteristics^∗^	HSPR%			*p* value
	Low (<3.5%)	Moderate (3.5%–10%)	High (≥10%)	
	No. 20	No. 17	No. 20	
Age (years)	61.0 (54.2–71.5)	67.0 (52.0–72.0)	57.5 (48.8–75.0)	0.63
Age (years)				0.67
≤64	11 (55.0%)	7 (41.2%)	11 (55.0%)	
>64	9 (45.0%)	10 (58.8%)	9 (45.0%)	
Sex				0.002
Female	15 (75.0%)	9 (52.9%)	4 (20.0%)	
Male	5 (25.0%)	8 (47.1%)	16 (80.0%)	
Tumor size (cm)	4.5 (2.0–6.1)	5.0 (3.5–7.0)	6.8 (4.0–7.5)	0.069
Tumor size (cm)				0.089
≤5	14 (70.0%)	10 (58.8%)	7 (35.0%)	
>5	6 (30.0%)	7 (41.2%)	13 (65.0%)	
Tumor location				0.58
Gastric	15 (75.0%)	13 (76.5%)	12 (60.0%)	
Nongastric	5 (25.0%)	4 (23.5%)	8 (40.0%)	
GIST group				0.0001
gr_1-3	19 (95.0%)	15 (88.2%)	8 (40.0%)	
gr_4-6	1 (5.0%)	2 (11.8%)	12 (60.0%)	
Mitotic score				0.0009
0–5	18 (90.0%)	15 (88.2%)	8 (40.0%)	
>5	2 (10.0%)	2 (11.8%)	12 (60.0%)	
Strong HSPR (no items per DOI)	3.0 (1.8–5.0)	115.0 (60.0–147.0)	1108.5 (181.0–3355.8)	0.007
Strong nHSPR(no items per DOI)	0.0 (0.0–1.2)	11.0 (1.0–15.0)	74.5 (28.2–995.2)	0.008
Weak HSPR(no items per DOI)	410.0 (151.0–523.2)	1180.0 (593.0–1527.0)	5608.5 (3406.0–8379.5)	<0.0001
Weak nHSPR(no items per DOI)	137.0 (37.0–350.0)	266.0 (171.0–363.0)	1760.5 (1206.2–6697.0)	0.002
nHSPR%	0.4 (0.1–0.6)	1.3 (0.8–3.0)	11.6 (3.7–24.3)	<0.0001
HSPR%_minus_nonHSPR%	0.6 (0.3–1.3)	4.1 (3.5–6.1)	11.7 (9.1–26.9)	<0.0001
Total number of cells	29166.5 (25333.8–39117.8)	19287.0 (16470.0–21826.0)	25545.0 (19779.8–31890.5)	0.002
Total number of cells				<0.0001
<25,000	5 (25.0%)	16 (94.1%)	9 (45.0%)	
≥25,000	15 (75.0%)	1 (5.9%)	11 (55.0%)	

**Table 3:** The AUC analysis for the diagnostic power of Ki-67, cyclin D1, p21, and p27 according to GIST groups.

	AUC	95% CI	*p* value	Optimal cut-off value
Cyclin D1_HSPR	0.594	0.429–0.76	0.26	—
p21 HSPR	0.74	0.599–0.88	0.0008	29%
p27 HSPR	0.696	0.529–0.862	0.02	49%
Ki67 HSPR	0.913	0.828–0.997	<0.0001	16%

## Data Availability

The data used to support the findings of this study are available from the corresponding author upon request.

## Abbreviations

GIST: Gastrointestinal tumor
MC: Mitotic count
ESMO: The European Society of Medical Oncology
HSPR%: Hotspot-positive percentage ratio
nHSPR%: Not-hotspot-positive percentage ratio.

## References

[1] Miettinen M., Lasota J. (2011). Histopathology of gastrointestinal stromal tumor. *Journal of Surgical Oncology*.

[2] The ESMO/European Sarcoma Network Working Group (2012). Gastrointestinal stromal tumors: ESMO Clinical Practice Guidelines for diagnosis, treatment and follow-up. *Annals of Oncology*.

[3] Hu Y., Gu R., Zhao J. (2017). Prognostic significance of Ki67 in Chinese women diagnosed with ER^+^/HER2^−^ breast cancers by the 2015 St. Gallen consensus classification. *BMC Cancer*.

[4] Penault-Llorca F., Radosevic-Robin N. (2017). Ki67 assessment in breast cancer: an update. *Pathology*.

[5] Duffy M. J., Harbeck N., Nap M. (2017). Clinical use of biomarkers in breast cancer: updated guidelines from the European Group on Tumor Markers (EGTM). *European Journal of Cancer*.

[6] Lamarca A., Walter T., Pavel M. (2017). Design and validation of the GI-NEC score to prognosticate overall survival in patients with high-grade gastrointestinal neuroendocrine carcinomas. *Journal of the National Cancer Institute*.

[7] Panzuto F., Merola E., Pavel M. E. (2017). Stage IV gastro‐entero‐pancreatic neuroendocrine neoplasms: a risk score to predict clinical outcome. *The Oncologist*.

[8] Chen M., Yao S., Cao Q., Xia M., Liu J., He M. (2017). The prognostic value of Ki67 in ovarian high-grade serous carcinoma: an 11-year cohort study of Chinese patients. *Oncotarget*.

[9] Melling N., Kowitz C. M., Simon R. (2016). High Ki67 expression is an independent good prognostic marker in colorectal cancer. *Journal of Clinical Pathology*.

[10] Li P., Xiao Z. T., Braciak T. A., Ou Q. J., Chen G., Oduncu F. S. (2016). Association between Ki67 index and clinicopathological features in colorectal cancer. *Oncology Research and Treatment*.

[11] Min K. W., Kim D. H., Son B. K. (2017). A high Ki67/BCL2 index could predict lower disease-free and overall survival in intestinal-type gastric cancer. *European Surgical Research*.

[12] Wandler A., Spaun E., Steiniche T., Nielsen P. S. (2016). Automated quantification of Ki67/MART1 stains may prevent false-negative melanoma diagnoses. *Journal of Cutaneous Pathology*.

[13] Tretiakova M. S., Wei W., Boyer H. D. (2016). Prognostic value of Ki67 in localized prostate carcinoma: a multi-institutional study of >1000 prostatectomies. *Prostate Cancer and Prostatic Diseases*.

[14] Turkel Kucukmetin N., Cicek B., Saruc M. (2015). Ki67 as a prognostic factor for long-term outcome following surgery in gastrointestinal stromal tumors. *European Journal of Gastroenterology & Hepatology*.

[15] Cuylen S., Blaukopf C., Politi A. Z. (2016). Ki-67 acts as a biological surfactant to disperse mitotic chromosomes. *Nature*.

[16] Lindboe C. F., Torp S. H. (2002). Comparison of Ki-67 equivalent antibodies. *Journal of Clinical Pathology*.

[17] Ács B., Kulka J., Kovács K. A. (2017). Comparison of 5 Ki-67 antibodies regarding reproducibility and capacity to predict prognosis in breast cancer: does the antibody matter?. *Human Pathology*.

[18] Thakur S. S., Li H., Chan A. M. Y. (2018). The use of automated Ki67 analysis to predict Oncotype DX risk-of-recurrence categories in early-stage breast cancer. *PLoS One*.

[19] Plancoulaine B., Laurinaviciene A., Herlin P. (2015). A methodology for comprehensive breast cancer Ki67 labeling index with intra-tumor heterogeneity appraisal based on hexagonal tiling of digital image analysis data. *Virchows Archiv*.

[20] Besusparis J., Plancoulaine B., Rasmusson A. (2016). Impact of tissue sampling on accuracy of Ki67 immunohistochemistry evaluation in breast cancer. *Diagnostic Pathology*.

[21] Shui R., Yu B., Bi R., Yang F., Yang W. (2015). An interobserver reproducibility analysis of Ki67 visual assessment in breast cancer. *PloS one*.

[22] Zhong F., Bi R., Yu B., Yang F., Yang W., Shui R. (2016). A comparison of visual assessment and automated digital image analysis of Ki67 labeling index in breast cancer. *PloS one*.

[23] Røge R., Riber-Hansen R., Nielsen S., Vyberg M. (2016). Proliferation assessment in breast carcinomas using digital image analysis based on virtual Ki67/cytokeratin double staining. *Breast Cancer Research and Treatment*.

[24] Focke C. M., Decker T., van Diest P. J. (2016). Intratumoral heterogeneity of Ki67 expression in early breast cancers exceeds variability between individual tumours. *Histopathology*.

[25] Lu H., Papathomas T. G., van Zessen D. (2014). Automated Selection of Hotspots (ASH): enhanced automated segmentation and adaptive step finding for Ki67 hotspot detection in adrenal cortical cancer. *Diagnostic Pathology*.

[26] Lewitowicz P., Matykiewicz J., Koziel D., Chrapek M., Horecka-Lewitowicz A., Gluszek S. (2016). CD63 and GLUT-1 overexpression could predict a poor clinical outcome in GIST: a study of 54 cases with follow-up. *Gastroenterology Research and Practice*.

[27] Liang Y. M., Li X. H., Li W. M., Lu Y. Y. (2012). Prognostic significance of PTEN, Ki-67 and CD44s expression patterns in gastrointestinal stromal tumors. *World Journal of Gastroenterology*.

[28] Belev B., Brčić I., Prejac J. (2013). Role of Ki-67 as a prognostic factor in gastrointestinal stromal tumors. *World Journal of Gastroenterology*.

[29] Kemmerling R., Weyland D., Kiesslich T. (2014). Robust linear regression model of Ki‑67 for mitotic rate in gastrointestinal stromal tumors. *Oncology Letters*.

[30] Basilio-de-Oliveira R. P., Pannain V. L. N. (2015). Prognostic angiogenic markers (endoglin, VEGF, CD31) and tumor cell proliferation (Ki67) for gastrointestinal stromal tumors. *World Journal of Gastroenterology*.

[31] Zhao W. Y., Xu J., Wang M. (2014). Prognostic value of Ki67 index in gastrointestinal stromal tumors. *International Journal of Clinical and Experimental Pathology*.

[32] Głuszek S., Rylski R., Kot M. (2008). GIST - ryzyko nawrotu i rozsiewu na podstawie obserwacji własnych. *Gastroenterology Review/Przegląd Gastroenterologiczny*.

[33] Gluszek S., Kot M., Matykiewicz J. (2006). Wyniki obserwacji chorych na nowotwory zrębu przewodu pokarmowego (GIST) leczonych chirurgicznie. *Polski Przeglad Chirurgiczny*.

[34] Wiraszka G. R., Głuszek S., Kozieł D. (2014). Characteristics of gastrointestinal stromal tumours, diagnostic procedure and therapeutic management and main directions of nursing practice in gastrointestinal stromal tumours. *Contemporary Oncology*.

[35] Liu X., Qiu H., Zhang P. (2018). Ki-67 labeling index may be a promising indicator to identify “very high-risk” gastrointestinal stromal tumor: a multicenter retrospective study of 1022 patients. *Human Pathology*.

[36] Sugita S., Hirano H., Hatanaka Y. (2018). Image analysis is an excellent tool for quantifying Ki-67 to predict the prognosis of gastrointestinal stromal tumor patients. *Pathology International*.

[37] Agarwal A., Gopinath A., Tetzlaff M. T., Prieto V. G. (2017). Phosphohistone-H3 and Ki67: useful markers in differentiating dermatofibroma from dermatofibrosarcoma protuberans and atypical fibrohistiocytic lesions. *The American Journal of Dermatopathology*.

[38] Bedekovics J., Irsai G., Hegyi K. (2018). Mitotic index determined by phosphohistone H3 immunohistochemistry for precise grading in follicular lymphoma. *Applied Immunohistochemistry & Molecular Morphology*.

[39] Winther T. L., Arnli M. B., Salvesen Ø., Torp S. H. (2016). Phosphohistone-H3 proliferation index is superior to mitotic index and MIB-1 expression as a predictor of recurrence in human meningiomas. *American Journal of Clinical Pathology*.

[40] Nielsen P. S., Riber-Hansen R., Schmidt H., Steiniche T. (2016). Automated quantification of proliferation with automated hot-spot selection in phosphohistone H3/MART1 dual-stained stage I/II melanoma. *Diagnostic Pathology*.

[41] Villani V., Mahadevan K. K., Ligorio M. (2016). Phosphorylated histone H3 (PHH3) is a superior proliferation marker for prognosis of pancreatic neuroendocrine tumors. *Annals of Surgical Oncology*.

[42] Ozturk Sari S., Taskin O. C., Gundogdu G. (2016). The impact of phosphohistone-H3-assisted mitotic count and Ki67 score in the determination of tumor grade and prediction of distant metastasis in well-differentiated pancreatic neuroendocrine tumors. *Endocrine Pathology*.

[43] Dessauvagie B. F., Thomas C., Robinson C., Frost F. A., Harvey J., Sterrett G. F. (2015). Validation of mitosis counting by automated phosphohistone H3 (PHH3) digital image analysis in a breast carcinoma tissue microarray. *Pathology*.

[44] Uguen A., Conq G., Doucet L. (2015). Immunostaining of phospho-histone H3 and Ki-67 improves reproducibility of recurrence risk assessment of gastrointestinal stromal tumors. *Virchows Archiv*.

[45] Shin Y., Hyeon J., Lee B. (2015). PHH3 as an ancillary mitotic marker in gastrointestinal stromal tumors. *Journal of Pathology and Translational Medicine*.

